# Implications of Vitamins in COVID-19 Prevention and Treatment through Immunomodulatory and Anti-Oxidative Mechanisms

**DOI:** 10.3390/antiox11010005

**Published:** 2021-12-21

**Authors:** Juan M. Toledano, Jorge Moreno-Fernandez, María Puche-Juarez, Julio J. Ochoa, Javier Diaz-Castro

**Affiliations:** 1 Department of Physiology, Faculty of Pharmacy, Campus Universitario de Cartuja, University of Granada, 18071 Granada, Spain; juanmatd97@correo.ugr.es (J.M.T.); javierdc@ugr.es (J.D.-C.); 2 Institute of Nutrition and Food Technology “José Mataix Verdú”, University of Granada, 18071 Granada, Spain; 3 Nutrition and Food Sciences Ph.D. Program, University of Granada, 18071 Granada, Spain; 4 Instituto de Investigación Biosanitaria (IBS), 18016 Granada, Spain

**Keywords:** vitamins, SARS-CoV-2, COVID-19, nutrients, immunity, inflammation, oxidative stress

## Abstract

Since the appearance of the coronavirus disease 2019 (COVID-19) and its announcement as a global pandemic, the search for prophylactic and therapeutic options have become a priority for governments and the scientific community. The approval of several vaccines against SARS-CoV-2 is being crucial to overcome this situation, although the victory will not be achieved while the whole population worldwide is not protected against the virus. This is why alternatives should be studied in order to successfully support the immune system before and during a possible infection. An optimal inflammatory and oxidative stress status depends on an adequate diet. Poor levels of several nutrients could be related to an impaired immune response and, therefore, an increased susceptibility to infection and serious outcomes. Vitamins exert a number of anti-microbial, immunomodulatory, anti-inflammatory, and antioxidant activities, which can be of use to fight against this and several other diseases (especially vitamin D and C). Even though they cannot be considered as a definitive therapeutic option, in part owing to the lack of solid conclusions from well-designed clinical trials, currently available evidence from similar respiratory diseases may indicate that it would be rational to deeply explore the use of vitamins during this global pandemic.

## 1. Introduction

The world is facing one of the most important public health crises of recent decades. The cluster of pneumonia cases described in the late 2019 was discovered to be produced by a new virus, which is well-known now as the severe acute respiratory syndrome coronavirus 2 (SARS-CoV-2). It is responsible for the development of the coronavirus disease 2019 (COVID-19), which has rapidly spread to every country worldwide, to the extent that it has been proclaimed by the World Health Organization (WHO) as a global pandemic [1].

The new pathogen, i.e., the seventh known human coronavirus, belongs to a class of viruses (β-coronaviruses) which are common among mammals and birds. However, all of them have had a tremendous impact around the world as this novel pathogen [2]. Its genetic similarity to SARS-CoV, which produced the 2002 outbreak of acute respiratory distress syndrome (ARDS), gave this new coronavirus its name. Nevertheless, SARS-CoV-2 is completely unknown for the immune system, so that the lack of underlying natural immunity against it may be one of the causes of its quick spread [3].

It is an enveloped single-stranded and positive-sense RNA virus that mostly affects the respiratory tract. The main route of transmission is due to droplets generated by an infected person who coughs sneezes or breathes, and contact with mucus environment such as saliva or nasal fluids [4]. This virus enters the cell through the angiotensin-converting enzyme 2 (ACE2) receptor, whereby it mainly infects alveolar epithelial cells in the lower respiratory tract [5]. The clinical spectrum of COVID-19 is wide, since disease manifestations in the respiratory tract might be lacking (asymptomatic patients), mild, moderate, or severe. The most frequent symptoms are cold-like or mild influenza-like, including fever, coughing, chills, fatigue, and shortness of breathing. Other described symptoms have been chilblains, anosmia, and ageusia. However, severe cases are characterized by respiratory and extra-respiratory complications that include ARDS, acute cardiac and vascular complications, multiple organ disfunction, septic shock, and respiratory failure, which frequently require ventilatory support [6].

These complications are believed to be associated with uncontrolled inflammation owing to abnormally strong release of cytokines triggered by viral replication. This response might result in pulmonary tissue damage, reduced lung capacity, and functional impairment [6]. In fact, two phases of immune response have been described during SARS-CoV-2 infection. The first one is characterized by a defense-based protective activity, while the second one is represented by broad inflammation. Therefore, therapeutic management should be oriented toward strategies that enhance immunity in the first phase and suppress it in the second one. Stimulating the immune system in mild stages of the disease can be advisable; however, in contrast, once lung impairment and other complications have already appeared, immunosuppression would be required instead [7].

Due to the fast evolution of outbreaks, strict national policies have been established as mitigation strategies in order to control the disease, including face masks, social distancing, staying at home measures, expansive testing, and contact tracing. Although some therapeutic alternatives look promising, there is not solid and complete evidence of the efficacy and safety in human trials of any proposed treatment for the disease. As for prophylactic interventions, the development and distribution of vaccines is now a reality. However, complete vaccination of the whole population worldwide is a goal far from being reached yet [8].

This is why the knowledge of other possibilities to prevent the disease or improve its management becomes relevant. In this context, nutritional and lifestyle factors should be taken into consideration as a complement to vaccination or treatment, and also as a way to enhance immunity before coming in contact with the virus. Immune system activity is enhanced during infection, which is accompanied by an increased metabolism. This is translated in an augmented need for energy sources, substrates, and regulatory molecules [8]. Individuals frequently neglect optimal dietary patterns and healthy nutritional status, especially during this stressful time of restrictions. However, an adequate provision of several nutrients (even beyond the classic essential requirements) provided by both a balanced diet and supplementation, is fundamental for a suitable immune response and an optimal modulation of inflammation and oxidative stress [9]. Among these nutrients, vitamins should be specially taken into consideration, as they exert several mechanisms related to immune function and oxidative stress that could be beneficial during SARS-CoV-2 infection. This is supported by currently available scientific evidence related to similar respiratory infectious diseases and, in some cases, to COVID-19 itself, which will be addressed in the following sections.

## 2. Materials and Methods

Bibliographical research started in early March 2021 until the end of July 2021, when the current review was conducted. The search was focused on the period between 2019 and 2021, through the main biomedical sources and databases: Medline (via PubMed), Elsevier, The Cochrane Library, and Dialnet. Only relevant articles published in recent years were accepted, and all of them were related to the subject of this study. Attention was preferentially paid to those related to COVID-19 and the association that vitamins could have with it. The search was entirely made in English, as it is the lingua franca used in the scientific field. Key words applied to do so have been COVID-19, SARS-CoV-2, vitamins, vitamin D, vitamin C, vitamin A, vitamin E, vitamin B, nutrition, nutrients, inflammation, and oxidative stress. Medical subject heading (MSH) terms were used in the words that may create misunderstandings in the browser. Boolean operators applied were “AND”, “OR”, and “NOT”. They were combined with key words to find valuable articles for this review. “AND” was used between every word in order to provide the search with more sensitivity and specificity, “OR” was used to connect synonyms, and “NOT” was not frequently used to avoid confusion in the browser.

Inclusion criteria are systematic reviews, randomized controlled studies, meta-analysis, and animal model studies, written in English and free of payment, especially studies involving humans or animals with a clinical diagnosis of SARS-CoV-2 infection. Exclusion criteria include the absence of an abstract, and any language different from English.

**Figure 1:**
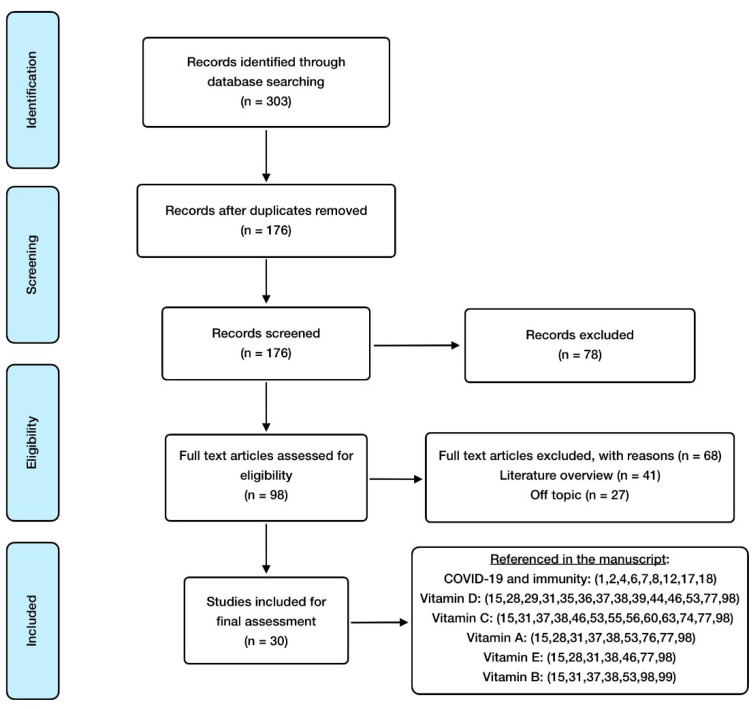
PRISMA flowchart summary for the manuscripts and scientific contribution selection.

Mendeley Reference Manager and Mendeley Desktop Library were used as the software for article management and bibliography organization. Regarding search methodology, the selection process carried out is detailed in Figure 1.

## 3. Results and Discussion

### 3.1. The Link between Immunity, Oxidative Stress and COVID-19

The development and consequences of an infectious disease were not only determined by the responsible pathogen. Immune system state and oxidative stress have a crucial influence that can modify the outcome of the infection [10].

In order to enter the organism, microbes need to go through natural barriers that protect the body against them. This is why adequate membrane state and junction integrity in epithelia is so important, so as to prevent pathogens from invading tissues, i.e., something that can be enhanced by vitamins such as D or C. Mucosal IgA also has a major implication in protecting these physical barriers against viruses, to the extent that an increased IgA response has been observed in severe cases of COVID-19 [11].

Once pathogens manage to overcome this first line of defense, they must face both innate and adaptive immunity, which tries to neutralize them. Although the precise interactions between SARS-CoV-2 and the immune system have not been fully elucidated yet, the cells involved in it seem to have a completely relevant role in the clinical symptoms and severity of the disease. This assumption is also based on previous studies performed with its closest relative, SARS-CoV [12]. The response starts with the recognition of pathogen-associated molecular patterns (PAMPs) by pattern-recognition receptors (PRRs), which triggers signaling pathways that lead to the expression of inflammatory mediators [13]. Coronaviruses are known for encoding multiple proteins that interfere with this recognition process and the subsequent activation of viral-controlling mechanisms. In particular, they have the ability of blocking viral RNA recognition and IFN-I responses through viral M protein action [14]. Vitamins C and A can be relevant in order to mitigate this detrimental viral activity.

Primary inflammatory response to SARS-CoV-2 comes with the release of several cytokines (including interleukin 6 (IL-6) and IL-1β) and tumor necrosis factor alpha (TNFα). They mediate inflammation through stimulation of several receptors, which activate intracellular signaling pathways and mediators, such as nuclear factor kappa-B (NF-κB) [15]. In this sense, and with the purpose of getting an optimal immune response, a certain level of inflammation is physiologically required after the exposure to a microbe. Nevertheless, a chronic state of low-grade systemic inflammation is associated with a dysregulation of the immune system, and, as a result, an increased risk of infection [16]. This situation is a common feature of several illnesses, such as cardiovascular disease (CVD), type 2 diabetes (T2D), metabolic syndrome, overweight/obesity, arthritis, or cancer. All of them are considered as comorbidities for COVID-19 patients [16]. Within this context, extreme conditions triggered by COVID-19 may generate several issues for infected patients, especially if they also suffer from these diseases.

In fact, excessive inflammation due to viral replication is considered the main cause of complications associated with the severe pneumonia produced by coronaviruses [17]. The infection causes an augmented release of several cytokines, chemokines, and other related molecules, such as IL-1β, IL-10, IL-4, interferon gamma (IFN-γ), interferon inducible protein 10 (IP-10), monocyte chemoattractant protein 1 (MCP-1), TNFα, IL-2, IL-7, and granulocyte colony-stimulating factor (G-CSF), which is specially observed in patients hospitalized in intensive care units (ICU). Furthermore, it has been reported that bronchial epithelial cells react to SARS-CoV and SARS-CoV-2 infection by generating an increased amount of NF-κB-mediated cytokines, such as IL-6 and IL-8 [17]. High production of IL-6 is associated with a deterioration in patients’ recovery, and may explain the elevated serum levels of C reactive protein (CRP), which are frequently lacking in viral infections [12].

As a consequence, this situation, known as “cytokine-storm”, results in hyper-inflammation, immune cell infiltration, edema, and excessive reactions, which can lead to life-threatening complications, such as hyperpyrexia, immune exhaustion, thrombosis, ARDS, and multiple organ failure [17]. This severe COVID-19-related cytokine profile has been undoubtedly found to correlate with poor therapeutic outcomes [18]. In conclusion, findings suggest that the inflammatory response generated in SARS-CoV-2 infection would be more destructive than the direct activity of the virus [12].

With regard to other immune cells, some peculiarities have been reported in COVID-19. Of note, exhaustion and reduced activity of NK cells and cytotoxic CD8+ T cells have been observed, especially at the early stages of the infection, leading to a more severe disease progression. The count of these cells has also been reported to be lower and, in some cases, also for memory CD4+ and regulatory T cells. The increased pro-inflammatory cytokine profile is also related to depletion and functional alteration of T cells [19,20].

In a similar way to the other coronavirus, SARS-CoV-2 is able to restrain antigen presentation by downregulating major histocompatibility complex (MHC), which attenuates T-cell-mediated immune responses. However, humoral immunity also plays a part in COVID-19, helping to neutralize the virus. Antibodies produced by B cells after infection can prevent the virus from entering the host cells and play a critical role in virus clearance [19]. Additionally, lymph nodes and spleen of COVID-19 patients were described as atrophic, a fact that points out the role of SARS-CoV-2 in cell degeneration [20]. Taken together, these findings highlight the immunosuppressive activity that SARS-CoV-2 exerts on the adaptive immune system. The majority of vitamins addressed in this review exert mechanisms of action that can be helpful in reducing cytokine storm intensity or ameliorating immune cell function.

It cannot either be underrated the role of oxidative stress, a situation is characterized by an imbalance between reactive oxygen (ROS) and reactive nitrogen (RNS) species, and the antioxidant systems (vitamin C, vitamin E, reduced glutathione and endogenous enzymes, such as superoxide dismutase (SOD), catalase (CAT), or glutathione peroxidase (GPx)) [15]. Excessive extracellular free radicals can oxidize biomolecules, such as proteins, lipids, DNA or RNA, and can modify the structure of proteins and genes that can trigger signaling pathways associated with inflammatory response [21]. The close relationship between inflammation and oxidative stress is widely known. Free radicals produced in the site of infection by immune cells, especially macrophages, are intended to neutralize invading pathogens [22]. However, chronically augmented oxidative stress, which appears in long-term infections, is related to impaired immune responses, augmented inflammation, and appearance of endothelial damage, i.e., situations which have been reported to play a vital role in COVID-19 prognosis [23].

Derived from SARS-CoV-2 infection produced in the organism, lung histology and function are seriously affected. The most commonly reported finding in COVID-19 patients’ histopathology has been diffuse alveolar damage (DAD), which is a manifestation characterized by the presence of hyaline membranes, interstitial edema, and fibroblast proliferation. Other pathologic features of DAD include enlarged and atypical type II pneumocytes, thrombi in small pulmonary arteries (derived from endothelial damage), and squamous metaplasia. More sporadic pathologic findings reported on COVID-19 are viral inclusions, intra-alveolar fibrin, and endotheliitis [24].

There are several physiological aspects that are interrelated with the immune system, including hormonal and metabolic regulation, circadian rhythms, and nutrient utilization. Broadly speaking, malnutrition can significantly compromise the immune response, as it may alter proliferation and activity of immune cells, making patients more prone to infection [25]. Moreover, inflammation related to poor dietary habits is reaching proportions never observed before, which is related to the coexistence of a wide range of non-communicable diseases (NCDs). These comorbidities may intensify and aggravate the inflammatory pathology and increase the risk of complications and mortality. For instance, it is known that people at high risk of CVD are also at greater risk of severe COVID-19, owing to the augmented ACE-2 expression observed in them [26].

Dietary modifications and nutritional factors, including fulfilling the recommended daily quantities of each nutrient through food or supplementation, can strongly counteract this chronic low-grade of inflammation, reducing the risk of getting an infection, such as COVID-19, and, of course, decreasing the likelihood of a fatal outcome [15]. This is proved by an increasing body of evidence, suggesting that this path may be the best for strengthening the immune system and preventing both inflammation and oxidative stress. It is presumed that a proper immune response in asymptomatic patients avoids progression to a severe stage in the disease, so strategies focused on boosting immunity could be defining in early stages of the infection. In addition, some nutrients may also come in handy in the management of COVID-19 as an adjuvant therapy, possibly developing additional pathways such as interaction with the cellular receptor or another important protein in the viral life cycle [7].

The following sections review the roles of vitamins D, C, A, E, and B in immunity, inflammation, and oxidative stress, and establish their relevance in order to prevent infection during the COVID-19 crisis, ameliorate the course of the disease if it takes place, and even promote healthy habits in these times of difficulties and restrictions. Scientific evidence about this subject and vitamin K is lacking, so it was not included in the review.

### 3.2. The Role of Vitamin D

#### 3.2.1. Vitamin D Mechanisms of Action

Its active form (1,25-dihydroxyvitamin D or calcitriol) is mainly known for regulating calcium homeostasis and bone health. However, it has also shown to play a role in the regulation of the immune system, so much that its deficient consumption is related to malfunction and dysregulation of immunity and inflammatory status [27].

**Figure 2:**
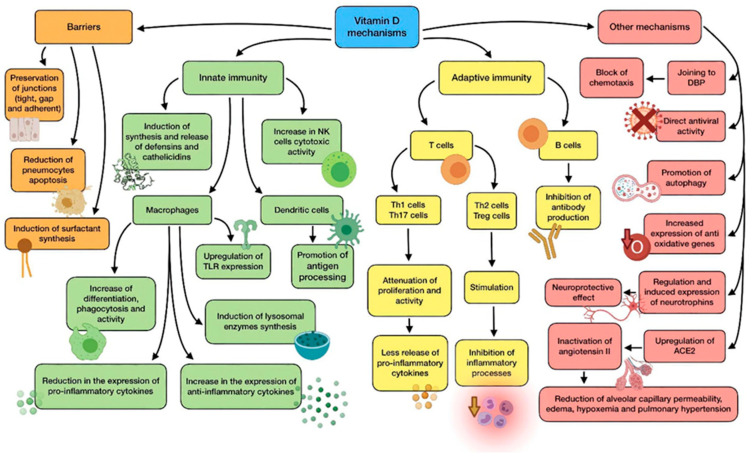
Summary of vitamin D main mechanisms of action.

Many studies suggest that vitamin D improves immune function, reducing susceptibility to infection. In contrast, an extensive number of scientific studies highlight its immunosuppressive effects. Thus, it seems that vitamin D supports immune response under physiological conditions, but it also has an active role in autoimmunity prevention. In short, its effects would depend on the immunological situation of the patient (health, infection, or autoimmune disease) [28]. A summary of vitamin D main mechanisms of action is shown in Figure 2.

The physiological link between vitamin D and the functioning of immune cells, such as lymphocytes, dendritic cells, and monocytes/macrophages is highly dependent, as they are able to carry out and enhance their functions after binding to calcitriol. In order to do so, these cells express CYP27B1 (1α-hydroxylase), which is responsible for vitamin D activation through the transformation of calcidiol (pre-vitamin D), into calcitriol [15].

Calcitriol interacts with the vitamin D receptor (VDR), a specific nuclear receptor which is mostly renowned for its role in the regulation of phosphorus and calcium levels, although it plays a part in both innate and adaptive immune systems. It is expressed by the previously cited immune cells, whose correct functioning, proliferation, and differentiation, therefore, depends on the bioavailability of the vitamin in them, due to this autocrine and paracrine mechanism. Airway epithelial cells also express CYP27B1, thus cooperating in the activation of immune cells. After attaching to vitamin D, activated VDR dimerise and the complex translocate to the nucleus so as to interact with regulatory sequences (VDRE) of target genes, modifying their transcription [29].

Vitamin D shows a great variety of mechanisms by which it protects against microbial infection, possibly reducing the risk of poor prognosis and death. They can be classified into three general categories: maintenance of physical barriers, improvement of innate immunity, and enhancement of adaptive immunity [30].

(a) Barriers: junction integrity may be disturbed by viruses, making invasion easier for other pathogens. In this field, vitamin D helps to preserve tight, gap, and adherent junctions between epithelial cells, reinforcing natural barriers against microorganisms [31].

Impairment in type II pneumocytes leads to a reduction in surfactant production, which increases the risk of ARDS. Vitamin D has proved to reduce pneumocyte apoptosis and induce surfactant synthesis, which would prevent serious lung injuries [32].

(b) Innate immune system: vitamin D is able to induce synthesis and release of antimicrobial peptides, such as cathelicidins (LL-37), and defensins in monocytes/macrophages [29]. Cathelicidins have demonstrated direct antimicrobial properties against a wide range of microbes, such as Gram-positive and Gram-negative bacteria, enveloped and non-enveloped viruses, and fungi. They reach these effects by altering cell membranes of infecting pathogens, as observed in a mouse model in which influenza A virus replication was reduced by cathelicidin LL-37 [33]. This protein has several other functions against microorganisms, including chemotaxis stimulation in neutrophils, monocytes and T cells, production of different cytokines, and induction of autophagy in infected epithelial cells (which boost the clearance of respiratory pathogens) [34]. As for defensins, β-defensin-2 also stimulates the expression of cytokines and chemokines related to the recruitment of immune cells. In the same way as cathelicidins, defensins are also able to block viral entry into cells [35].

In monocytes, vitamin D promotes differentiation to macrophages, increases phagocytosis, and increases superoxide output. Moreover, it can reduce the expression of pro-inflammatory cytokines and upregulate the expression of the anti-inflammatory ones, thus attenuating cytokine storm. Specially, vitamin D is able to reduce IL-6 expression through the p38 MAP kinase signaling pathway. Vitamin D also induces the synthesis of lysosomal enzymes and the release of NO, which cooperate in countering infection [9]. In dendritic cells, it promotes antigen processing, though it also might impair cell maturation and antigen presentation. In addition, vitamin D also upregulates the expression of TLR2 and TLR4, both in monocytes and dendritic cells. The interaction of molecules with the components of pathogens enhances the expression of CYP27B1 and VDR, in order to improve vitamin D-derived actions [29,36]. Besides, NK cells activity may be diminished by low-serum vitamin D, while high serum levels could significantly increase its cytotoxicity [36].

(c) Adaptive immune system: with regard to adaptive immunity, vitamin D modifies a response toward a more anti-inflammatory pattern, developing a different effect depending on the type of T cell. On the one hand, it has shown to attenuate Th1 cell proliferation and activity, which leads to a decrease in the release of pro-inflammatory cytokines that contribute to ARDS, such as TNFα, INF-γ, IL-6, and IL-2. On the other hand, vitamin D seems to stimulate Th2 cells and Treg cells, thereby inhibiting inflammatory processes. It also reduces Th17 activity and IL-17 production. Its impact on CD8+ T cells appears to be minor [37,38].

During the elicitation phase at the beginning of inflammation, vitamin D inhibits Th1, Th17, and the abnormal release of their cytokines. However, during the resolution phase, it induces Th2, Treg, and the release of theirs (specially IL-10), thus avoiding organ damage due to an excessive cytokine secreting response. In short, vitamin D promotes a balanced T cell’s defensive response against microbes [36]. As for B lymphocytes, vitamin D has also been reported to inhibit antibody production [38].

(d) Other relevant mechanisms: it has been proven through animal models that vitamin D attenuates ARDS-related to microbes by modulating the activity of the renin–angiotensin system (RAS) and the expression of angiotensin converter enzyme 1 and 2 (ACE1, ACE2). The increase in alveolar capillary permeability is one of the key mediators in this pathology, leading to pulmonary edema, pulmonary hypertension, and hypoxemia [31]. As ACE2 inactivates angiotensin II, it acts as a negative regulator of the RAS. Therefore, it may have a beneficial role in regulating vascular permeability and its pulmonary consequences during ARDS development. Calcitriol has demonstrated its ability to upregulate pulmonary ACE2 and downregulate angiotensin II and renin, so it can play a key role in hindering the progression of respiratory distress induced by infection [37]. This mechanism seems to clash somehow with SARS-CoV-2 characteristics, as it needs ACE2 to enter epithelial cells. Nevertheless, the binding of the S1 spike protein to ACE2 causes both the enzyme and the virus to be translocated into the cell, thus decreasing its surface expression and probably contributing to the development of pulmonary pathology. There seems to be an association CVD between high levels of ACE2 and survival likelihood, which in the end would be beneficial in ARDS and COVID-19 [31,39]. Furthermore, vitamin D deficiency induces activation of RAS, which may lead to CVD and reduced lung function, as well as comorbidities associated with a higher risk of severe cases of COVID-19 [39].

Vitamin D binding protein (DBP) is the main transport protein of this compound (though it can also bind actin and other molecules), and it might have an underrated role in the onset of ARDS on account of SARS-CoV-2 infection [37]. During ARDS, actin is released into extracellular fluids, and has a pro-coagulant action when it polymerizes from globular actin (G-actin) to filamentous actin (F-actin). As a response, DBP slices the F-actin actin and avoids G-actin repolymerization. However, DBP attached to G-actin acts as an indirect but relevant factor in inflammation by increasing the action of neutrophil chemoattractants. According to this, vitamin D would block chemotaxis by competing for the same binding site on DBP, so low levels of the vitamin could be correlated with a more serious outcome during infection [37].

The powerful neuroprotective effect of vitamin D should also be taken into consideration, as it could come in handy to prevent neurological symptoms produced by COVID-19. This effect is linked to regulation of neurotrophins, due to the fact that vitamin D stimulates expression of nerve growth factor (NGF), brain-derived neurotrophic factor (BDNF), and neurotrophin 3 (NT3), just like neurotrophin receptor p75NTR in neurons, glial cells, and Schwann cells. Ultimately, this is translated into protection against brain ischemia and neurodegenerative disorders. In addition, vitamin D has been reported to enhance remyelination of neurons by promoting the migration and differentiation of oligodendrocytes’ progenitors [40,41].

The vitamin has shown antiviral functions against a number of different microbes, including influenza virus or Epstein-Barr virus (EBV). The mechanisms involved in these functions are the production of antimicrobial peptides, blockage of viral entry, replication and induction of autophagy, induction of virus specific CD8+ T cells, and suppression of TLRs (different from TLR2 and TLR4) [36].

As for oxidative stress, vitamin D has been reported to upregulate the expression of some antioxidant genes (for example, glutathione reductase), thus reducing the amount of ROS generated because of inflammation which could contribute to ARDS and lung damage [31]. Excess of ROS production enhances NF-κB expression in immune cells, causing a raised production of pro-inflammatory cytokines and other mediators. According to this, ROS reduction promoted by vitamin D leads to a decrease in NF-κB pathway and, consequently, the genes regulated by it [29,37].

Promoting autophagy is another relevant mechanism of vitamin D against infection, as it is an essential way for cells to deal with viruses, thus enabling their degradation and subsequent antigen presentation. The specific mechanism involves downregulating the mTOR pathway (which inhibits autophagy), and upregulating Beclin-1 (BECN1) and class III phosphatidylinositol 3-kinase (PI3KC3), i.e., the key drivers of the process. As stated before, cathelicidin expression enhanced by vitamin D also induces this process [35].

Another noteworthy fact is that correction of vitamin D insufficiency significantly reduces the expression of dipeptidyl peptidase 4 (DPP-4 or CD26). This is one of the adhesion proteins through which SARS-CoV-2 is believed to get access to host cells, owing to an interaction with the S1 domain of spike glycoprotein. This suggests that it may be a prominent virulence factor in the disease [39].

#### 3.2.2. Evidence about Vitamin D Regarding Respiratory Infections and COVID-19

Vitamin D deficiency is widely common, affecting over a billion people worldwide. Among general population, there are two groups at particular risk: individuals with darker skin and the elderly. As for the first group, increased pigmentation complicates the penetration of UV light needed for epidermal synthesis (which represents the main source of vitamin D) [36]. This might be one of the factors involved in the disproportionate increase in COVID-19 affectation among black people compared to the rest of population [42]. Besides, a similar influence was observed in studies related to the 1918–1919 influenza pandemic [43]. With regard to elder people, a combination of factors (such as reduced epidermal synthesis, increased time indoors, reduced food intake and impairment in vitamin D metabolism owing to drugs) elevates their risk of suffering from vitamin D deficiency. Needless to say, the elderly has also been the group of population most severely affected by SARS-CoV-2 infection. Researchers have highlighted the relevance for vulnerable groups to maintain adequate vitamin D levels in order to reduce the risk of respiratory tract infections, including COVID-19 [15,44]. However, this pandemic has also increased the risk for general population to develop a vitamin D deficiency, due to lockdown or “staying at home” mitigation strategies, which are adopted to prevent the virus propagation of the virus. This reduced sun exposure time makes it undoubtedly more difficult to reach an adequate vitamin D status, especially during autumn and winter.

Another noteworthy fact is that countries have experimented on a more complicated situation with regard to the pandemic during and right after seasons with less sun exposure (autumn and winter), rather than after seasons with more sun hours (spring and summer). This might be connected to the lower vitamin D concentrations that population has in those periods of time. It has been suggested that latitude may play a role as well [45]. Though the recommended dietary allowance (RDA) of vitamin D is 15–20 µg/d, scientific evidence recommends doses of 250 µg/d for a month in order to quickly raise concentrations, followed by a maintenance dose of 125 µg/d for 12 months. Higher doses would be required for vulnerable groups and cases of present infection. According to their sun exposure, supplementation in countries far from the equator could be proposed as a routine without having to check plasma levels [46].

A meta-analysis of several randomized controlled trials (RCTs) showed an overall protective effect against ARDS due to vitamin D supplementation, with the most important benefits appearing in patients with a greater deficiency of this nutrient [47]. Some meta-analyses found a strong association between low vitamin D status and risk of respiratory tract infection, even though others have not achieved the same conclusion. Consequently, the usefulness of vitamin D supplementation in viral respiratory diseases remains unclear [48].

This vitamin has been discussed specifically for its role in influenza therapy and prevention, with mixed effects attained. A Chinese RCT in which vitamin D was administered to infants reported protective effects with regard to incidence and severity of the infection. Similar results were obtained by another RCT in Japan [49,50]. In addition, systematic reviews and meta-analyses on the role of this nutrient in influenza vaccination showed low seroprotection rates in those who had vitamin D deficiency. On the other hand, some studies fail to find a correlation between supplementation and reduction in infections, although they do not demonstrate a complete inefficacy for this intervention [51].

These findings related to respiratory tract infections have led to the speculation that vitamin D may have a protective role in COVID-19 [52]. There is some discrepancy regarding vitamin D deficiency as an independent risk factor for the disease, as poor prognosis would possibly be due to a combination of factors, among which vitamin D hypovitaminosis would be included. This situation is likely in individuals with obesity, hypertension, or diabetes, i.e., three risk factors linked to greater severity of SARS-CoV-2 infection [53].

A retrospective observational study showed that infected subjects had a more severe vitamin D deficiency than control subjects [37]. Another retrospective study reported worse outcomes in the disease for those patients with vitamin deficiency [29]. Adequate status has been associated with a 15% reduction in severe cases among the elderly. Ilie et al. [54] suggested a possible correlation between vitamin D status and incidence and mortality because of COVID-19, as they found particularly low levels in the aging population of the countries which had been more affected by the pandemic.

Even though there is information and knowledge pointing to a prophylactic and/or therapeutic value of vitamin D against COVID-19, there is a lack of conclusive data, so no final assertion can be made yet. Further research and trials are required so as to completely demonstrate this hypothesis [44]. Nevertheless, it can be advisable for the general population to keep adequate levels of this vitamin, either by sun exposure, diet, or supplementation, especially in the most vulnerable groups. This would be an easy, cheap, and safe option that could potentially help reduce the burden generated by the pandemic. When it comes to supplementation, interventions needs to be controlled, as high doses may lead to the appearance of hypercalcemia [36,53].

### 3.3. The Role of Vitamin C

#### 3.3.1. Vitamin C Mechanisms of Action

Vitamin C (also known as ascorbic acid) is a nutrient required as a cofactor for multiple enzymatic reactions, such us norepinephrine biosynthesis, collagen hydroxylation, or amidation of peptide hormones. Additionally, it exerts a wide range of properties that could come in handy for the prevention and treatment of infections, including antioxidant, immunomodulating, as well as antiviral and antithrombotic functions [55].

**Figure 3:**
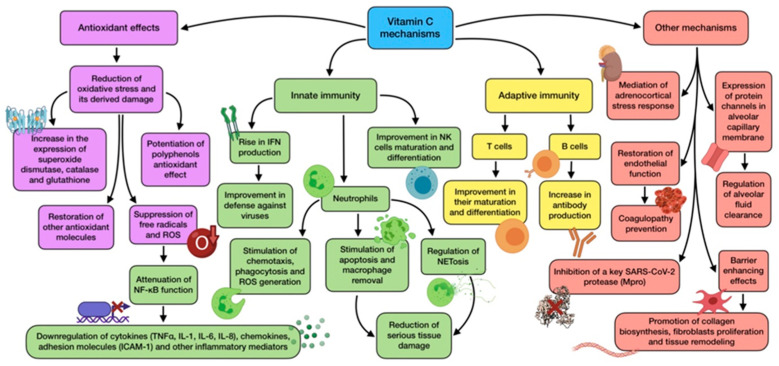
Summary of the main vitamin C mechanisms of action.

Vitamin C is mostly renowned for being a classical and potent antioxidant, quenching free radicals while being transformed into its oxidized form (dehydro-ascorbic acid). It also helps to restore other antioxidant molecules, such as vitamin E and tetrahydrobiopterin. Furthermore, it may potentiate the pharmacological effects carried out by flavonoids (for example quercetin), so an association between them could possibly exert a valuable synergistic antioxidant activity through a combination of the scavenging mechanisms performed by flavonoids and the transcription-modulating and scavenging mechanisms performed by ascorbic acid. However, this is only theoretical, so further investigation would be needed to clarify the usefulness of this combination in vivo [56]. The mainvitamin C mechanisms of action are summarized in Figure 3.

Oxidative stress is a frequent feature during infection and inflammation, owing to the release of ROS from activated phagocytes as part of the host response to pathogens. As for COVID-19, neutrophil-derived oxidative stress is thought to produce tissue damage [57]. Vitamin C optimal status is associated with less oxidative stress, has been reported to repair oxidative damage in bronchial epithelium, and is also able to prevent ROS-induced lung damage [58]. Ascorbic acid antioxidant role interacts with its immunomodulatory activity through nuclear transcription factor NF-κB. The reduction in ROS promoted by vitamin C attenuates NF-κB function, thus avoiding transcription of pro-inflammatory genes regulated by it. This includes the ones that encode TNFα, IL-1, IL-6, and IL-8; some chemokines; adhesion molecules (ICAM-1); and other inflammatory mediators. This leads to a downregulation of inflammatory cytokine production and, as a consequence, a mitigation in the severity of cytokine storm triggered by ARDS [55]. In addition, epigenetic regulation of genes by vitamin C also affects human natural antioxidant mechanisms, such as superoxide dismutase, catalase, and glutathione, whose expression seems to be increased by this nutrient [55,59].

A proper vitamin C status plays a role in immunity (both innate and adaptive) and host susceptibility to infection, as it accumulates in leukocytes and maintains their normal functioning, especially in neutrophils [60]. It has not only proved to stimulate neutrophil chemotaxis and migration to the site of infection, but also their phagocytic function and ROS generation [55]. Moreover, vitamin C is able to support neutrophil apoptosis and enhance macrophage removal, avoiding serious damage in host tissue [61]. This vitamin has also been reported to have a role in regulation of neutrophil extracellular traps (NETosis). This is a cell death pathway, distinct from apoptosis and necrosis, whose objective is the inactivation of pathogens. However, an excess in this response is considered a maladaptive feature which contributes to tissue damage and organ failure, as implicated in COVID-19 thrombotic complications. Ascorbic acid deficiency in septic animals has shown to increase plasma cell-free DNA generated from NETosis, so administration could attenuate this detrimental process [62,63].

Vitamin C is also related to other immune cells, including T and NK cells, whose maturation and differentiation is promoted by this nutrient [64]. Ascorbic acid-deficient diet has been reported to decrease vitamin C cell content, reducing T cell activity and ability to recall antigens [38]. It is also involved in antibody production by peripheral blood B cells. Age-related vitamin deficiency is associated with low serum levels of IgM and IgG, thus presenting a situation that would be reverted with supplementation [38,60].

One of the most important antiviral effects that vitamin C exerts is related to its ability to rise IFN production. While SARS-CoV-2 downregulates IFN expression and release, ascorbic acid is able to enhance it, thus allowing this antiviral cytokine to carry out its key defensive role against viruses [63]. Vitamin C also shows several barrier-enhancing effects that cooperate in maintaining epithelial integrity. Collagen biosynthesis requires participation, and is only negatively affected by severe vitamin C deficiency. This vitamin can also modify gene expression in dermal fibroblasts, promoting their proliferation and, therefore, tissue remodeling [15].

There is also increasing evidence which points out that vitamin C might play a critical role in mediating the adrenocortical stress response. Adrenal glands have three times more vitamin C concentration than any other organ, and are released from the adrenal cortex during physiological stress situations, such as viral exposure. The increased vitamin levels enhance cortisol production and potentiates anti-inflammatory and endothelial-protective effects carried out by glucocorticoids [65]. Furthermore, vitamin C may also be beneficial with regard to another common issue in COVID-19, known as coagulopathy. As vitamin C is able to restore endothelial function, it would help decrease the risk of these complications. Early injection has shown to prevent microtrombi formation and capillary plugging [62]. Likewise, lower concentrations of tissue plasminogen activator (tPA) and C reactive protein have been related to an increased dietary vitamin intake [15].

On account of all these mechanisms and functions, and through many in vitro studies, animal experiments, and clinical trials, vitamin C has been reported to exhibit antiviral properties against a wide range of microbes involved in several diseases, including influenza, common cold, and even coronavirus [37]. Pre-clinical data show that ascorbic acid may have a direct antiviral effect against both RNA and DNA viruses. Nevertheless, if vitamin C also has this effect against viral replication in vivo is yet to be confirmed [60]. With regard to SARS-CoV-2, the possible relationship between a key virus protease (M-pro) and vitamin C has become noteworthy, as an in-silico study revealed that the active site of this enzyme has the strongest binding with magnesium ascorbate, out of 106 nutraceuticals. Therefore, this evidence suggests that ascorbate may be a powerful enzyme inhibitor [66]. Despite the acknowledged properties of vitamin C, whether or not they are translated into a real beneficial effect in the response to COVID-19 remain to be elucidated, even though findings point to a significant potential to ameliorate negative consequences of SARS-CoV-2 infection and possibly become a feasible treatment option [31].

#### 3.3.2. Evidence about Vitamin C Regarding Respiratory Infections and COVID-19

There is a belief that vitamin C helps to prevent and treat upper respiratory tract infections (URT). However, the dose needed (1–2 g/d) is unattainable from diet, so supplementation might be recommended [60].

Vitamin C consumption has been a common practice during cold or flu for decades, partly owing to research published by Linus Pauling in 1970 about RCTs regarding this subject [67]. More recent meta-analysis of several studies concluded that vitamin C low-dose supplementation does not decrease the risk of contracting a cold, but high doses exhibit other benefits, including a reduction in cold severity and symptoms, just as a reduction in duration and recovery time [68]. This vitamin also seems to provide a greater resistance to viral infections during exposure to physical and physiological stress, halving their incidence and conferring a prophylactic benefit [31,68]. The similarity of symptoms and the positive effect of ascorbic acid across a number of cold-related viruses has led to hypothesize that vitamin C’s activity is not virus-specific, and would, therefore, alleviate COVID-19 symptoms [55].

Vitamin C deficiency and its related disease scurvy have long been associated with increased susceptibility to pneumonia, which can be reversed by supplementation, with a particular benefit for individuals with lower dietary intakes [37]. This has been reported by different RCTs whose subjects significantly reduced pneumonia incidence when oral vitamin C was administered [69]. A decrease in the duration of hospital stay for those receiving earlier and higher doses of vitamin C has been reported [60].

Low oral bioavailability and downregulation of sodium-dependent vitamin C transporter 2 (SVCT2) by inflammatory cytokines led to the hypothesis that vitamin C therapeutic levels needed to mitigate oxidative stress in critically ill patients cannot be attainable through oral administration [62]. As for these individuals (suffering from burns, ARDS, sepsis, and septic shock), intravenous administration of ascorbic acid has been tested, and results highlight lower mortality according to meta-analysis [70]. These patients have higher vitamin C requirements than general population in order to normalize their blood levels, which have been seriously reduced by their pathology [63]. In the largest trial related to this research, the CITRIS-ALI trial, patients receiving a high dose of intravenous vitamin C did not appreciably ameliorate organ dysfunction scores, markers of inflammation, or vascular damage, though mortality rate was dramatically reduced. In addition, a reduction in intensive care unit (ICU) stay and mechanical ventilation was reported. This will conceivably improve long-term outcomes, especially if they keep on taking the vitamin orally after ICU discharge [62,71]. In spite of these positive results, the intravenous use of vitamin C should be performed cautiously, as in some cases and depending on dosage, it might exert a pro-oxidant effect rather than antioxidant [72].

Even though the evidence gathered so far about the specific utility of vitamin C against SARS-CoV-2 infection is limited, some related findings deserve to be highlighted. For instance, despite ascorbic acid, suboptimal intake poorly correlates with the disease incidence and correlates in an appreciable way with deaths [53]. Studies carried out in the USA and Spain showed a state of hypovitaminosis among COVID-19 patients with ARDS, which was even lower between non-survivors. It is also interesting that many risk factors for vitamin C deficiency overlap with those for COVID-19 (male; older; darker skin; patients suffering from hypertension, diabetes, or chronic obstructive pulmonary disease (COPD)) [55]. Vitamin C has been included in the treatment of some COVID-19 ICUs patients, which have shown to improve mortality rate. Intravenous administration of high doses in China and USA exhibited promising results in patients with moderate to severe disease, as it attenuated cytokine storm during the late stage of the infection, just as lung inflammation and injury [60,73]. In addition, there are some case reports of COVID-19 patients that described less mortality, mechanical ventilation need, and a decrease in inflammatory markers when using intravenous vitamin C [74].

Low ascorbic acid levels are frequent in COVID-19 critically ill patients (its body store is depleted due to oxidative stress, which leads to an increased physiological demand). This fact, along with all the findings presented before, suggest that vitamin C supplementation would be advisable to restore regular levels, in order to both prevent and treat these condition, as adequate status cannot be attainable through dietary sources [46,60]. The RDA of vitamin C for healthy adults is 75–90 mg/d. Owing to the lack of evidence on COVID-19, recommendations for vitamin C intake are limited. Doses of 1–2 g/d have previously been effective in preventing upper respiratory infections or reducing their duration. However, this does not apply when the patient is suffering the infection, as they need bigger doses in order to restore depleted levels. As mentioned before, individuals with serious disease would require even higher intravenous doses, about 50 mg/kg/6 h administered for 4 days. Association with polyphenols, flavonoids, and anthocyanins should be taken into consideration, as they could have an important role [31,46].

The potential benefits of this vitamin, including its low cost and safety profile, make it an attractive candidate and warrant its use. Some adverse effects have been reported in high dose administration cases, although they are of little consequence and related to patients with underlying conditions [31,63]. Patients at higher risk of COVID-19 mortality and vitamin C deficiency should take this recommendation more seriously, as they are the ones who can experiment the most severe outcomes, and at the same time, the ones who can benefit the most [55]. However, carefully designed RCTs are still needed in order to accurately assess the role of the vitamin in this disease before supplementation can be considered as a standard of care [31].

### 3.4. The Role of Vitamin A

#### 3.4.1. Vitamin A Mechanisms of Action

**Figure 4:**
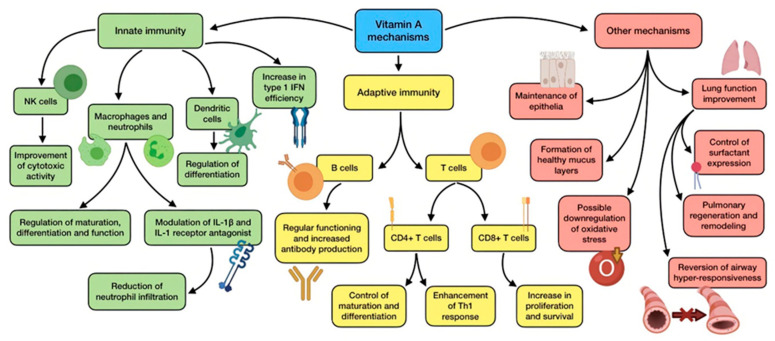
Summary of the main vitamin A mechanisms of action.

Its active forms retinal, retinol, and, especially, retinoic acid (RA), which mediate the transcription of hundreds of genes implicated in several biological pathways. They participate in many biological activities, such as vision maintenance, bone metabolism, as well as epithelial and membrane regulation, just as antioxidant properties. Nevertheless, this vitamin has also shown to play a key role in the modulation and regulation of the immune system, including innate and adaptive responses, which may enact a crucial element in the fight against pathogens [37]. Figure 4 summarizes the mechanisms of action of vitamin A.

With regard to innate immunity, vitamin A regulates the maturation, differentiation, and function of its main cells (macrophages and neutrophils). It also supports the rapid response against pathogen invasion by promoting phagocytosis and NK cells cytotoxic activity. Additionally, this vitamin is also associated with dendritic cell precursors, as it can alter their differentiation [75]. RA is also involved in the pathogenesis of ARDS, through a modulation in the production of IL-1β and IL-1 receptor antagonist by alveolar macrophages, and the following neutrophils infiltration [31]. Among the immunomodulatory properties exhibited by retinoids, their ability to increase the efficiency of type 1 interferon (IFN-I) is to be taken into consideration, due to its crucial role in the fight against viral infections. As coronaviruses are able to suppress the host antiviral response mediated by IFN-I, retinoids have been considered as a treatment option in order to fix this imbalance [76].

As for acquired immunity, the impact of vitamin A is not clear, and might be dependent on the setting and the vitamin metabolite involved. It controls the maturation and differentiation of CD4+ T cells (through an increase in IL-2 release), and experimental studies have indicated an enhancement in Th1 response promoted by RA [38]. This metabolite is also required for CD8+ T cell proliferation and survival, and has a role in antibody generation and normal functioning of B cells. This is the reason why it has been used as an adjuvant in vaccination against both bacteria and viruses, so as to improve antibody response [77]. Vitamin A is also important for epithelia maintenance, with a role in its differentiation, maturation, stratification and keratinization, thus reinforcing the front line of defense against microbes. Another benefit provided by this vitamin is the promotion of the formation of healthy mucus layers (including those in the respiratory and intestinal tract) [15]. According to all these facts, low vitamin A status and deficiency is associated with:

- Altered immune responses, both innate and adaptive [38];
- Hampered function of macrophages, neutrophils, T cells, and B cells [53];
- Increased number of neutrophils in blood (though their phagocytic function gets impaired) [38];
- Altered balance between Th1 and Th2 lymphocytes [38];
- Diminished function of NK cells, reducing antiviral defenses [28];
- Histopathological alterations (squamous metaplasia) of the pulmonary epithelium and lung parenchyma, resulting in an augmented risk of respiratory disease [78];
- Impaired barrier function, including deficient mucus secretion and breakdown of gut barrier [38];
- Lessened response to vaccination [28].

This would consequently lead to a situation of more susceptibility to a range of infections, just as an increase in morbidity and mortality derived from infectious diseases [79].

Vitamin A supplementation is also able to improve pulmonary function in patients with COPD and asthma. This has been explained and demonstrated by in vitro work and pediatric studies, which reported that retinoid acid was able to control the expression of surfactant protein, reverse airway hyper-responsiveness, and downregulate oxidative stress [31]. However, the oxidative ability of this vitamin has been subject to much debate, as it has been discussed for being both anti-oxidative as well as pro-oxidative depending on the case [80]. Animal studies have reported improved pulmonary regeneration and remodeling when a combination of RA and simvastatin was administrated. This not only caused oxidative damage, but also meant that the regenerative capacity of lungs might be partly mediated by vitamin A-dependent mechanisms. All these findings highlight the possible protective role of vitamin A in the pathogenesis of ARDS, a common complication in severe cases of COVID-19 [81].

Finally, retinoids have also exhibited direct inhibitory activity against a wide range of viruses, including influenza virus. In addition, retinoid signaling could powerfully block coronaviruses [37]. Through a library screen, Yuan et al. [82] found a specific agonist for RARα which acts as an inhibitor for both SARS-CoV and MERS-CoV viruses.

#### 3.4.2. Evidence about Vitamin A Regarding Respiratory Infections and COVID-19

Systematic reviews and meta-analyses have reported that retinoid administration improves symptoms related to acute pneumonia, and also reduces incidence, morbidity, and mortality of measles [83]. However, and in spite of all the mechanisms previously cited, there is a lack of direct clinical evidence linking vitamin A supplementation to enhanced resistance to infection [84].

There also seems to be a lack of association between vitamin A plasma levels and actual response to vaccination in the elderly. At large, studies investigating the usefulness of this vitamin with regard to the enhancement of immune response to vaccines have generated conflicting results [85]. Furthermore, available data have suggested that supplementation should not generally be advisable for lower respiratory tract infection prevention, as it was found out that administration did not affect their risk of appearance [86]. Nevertheless, these results might be influenced by the fact that low status of this vitamin is rather uncommon in westernized countries, together with the influence of pre-existing vitamin stores. The RDA of this vitamin for adults is set at 750 µg RAE/d (retinol activity equivalent), which is the same as 750 µg/d of dietary retinol. As it is unlikely to have a deficiency regarding this vitamin, supplementation should only be considered with great care, unless a parenteral diet is considered. This is why more controlled studies are needed in order to sufficiently clarify the topic [15,53].

However, a correlation between lower concentration of vitamin A and host susceptibility to influenza and COVID-19 has been found. Therefore, and even though there is still no clinical evidence regarding vitamin A and COVID-19, in the light of its roles in lung function and immunity, the vitamin is currently been investigated for the treatment of SARS-CoV-2 infection alongside with other antioxidants [31].

### 3.5. The Role of Vitamin E

#### 3.5.1. Vitamin E Mechanisms of Action

Among the multiple isoforms (four tocopherols and four tocotrienols) of this fat-soluble vitamin, α-tocopherol is the most recognized to meet human requirements.

**Figure 5:**
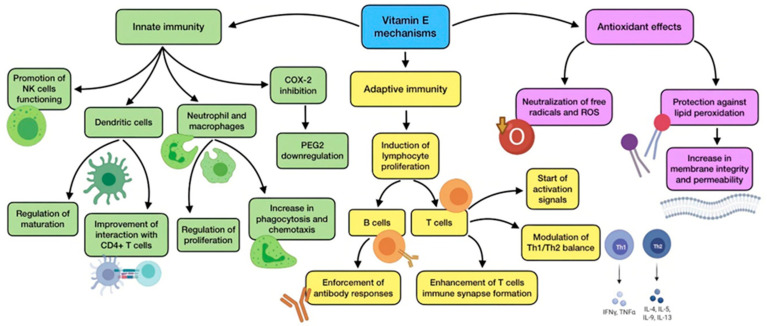
Summary of vitamin E main mechanisms of action.

The main activity of vitamin E is related to its potent antioxidant activity, which makes it capable of neutralizing free radicals and ROS, thus reducing oxidative stress. By doing so, vitamin E can protect polyunsaturated fatty acids (PUFAs) located in cell membranes from reacting with these species, avoiding peroxidative decomposition, loss of integrity, and increased permeability [46]. A summary of vitamin E’s main mechanisms of action is shown in Figure 5.

An inverse relationship between this vitamin levels and plasma lipoperoxidase has been clinically observed in ARDS patients, which supports the fact that vitamin E deficiency results in higher levels of lipid peroxidation [87]. Many people do not get enough vitamin E from dietary sources, even though its antioxidant effect could be beneficial during SARS-CoV-2 infection, as oxidant–antioxidant imbalance due to excessive oxidative stress and inflammation is one of the main pathological mechanisms that underlies the biology of COVID-19-derived ARDS. Animal ARDS models have also shown a significant improvement in lung compliance and gas exchange due to enteral α-tocopherol administration, also demonstrating dose-dependent effects related to vascular permeability and pulmonary artery pressure [31]. Of note, vitamin E works synergistically with vitamin C in order to enhance their antioxidant activity, i.e., α-tocopherol reduced by ascorbic acid [15]. In an animal study, vitamin E administration reached better results in reducing the altered oxidative status resulting from influenza infection compared to ascorbic acid. However, the combination of both was the intervention which most successfully reduced lipid peroxidation [88].

In spite of the importance of α-tocopherol, this is not the only isoform with relevant properties concerning respiratory diseases. Other tocopherols (and specially γ-tocopherol) studied in supplementation trials have also shown important roles, which may be different from α-tocopherol effects. While α-tocopherol is able to basically scavenge ROS, γ-tocopherol can neutralize both ROS and RNS. It has also demonstrated benefits on neutrophilic airway inflammation, and has shown to decrease several signs of acute lung injury, including impaired gas exchange, airway obstruction, and pulmonary edema. In addition, it reduces the need of mechanical ventilation in animal studies [89,90].

High concentrations of this vitamin are found in immune cells, conferring them protection from oxidative damage owing to their high PUFA content and metabolic activity. Likewise, vitamin E is also involved in the functioning of the immune system, showing immunomodulatory and anti-inflammatory effects. Numerous studies highlight that deficiency negatively affects both humoral and cell-mediated immunity [46]. This vitamin plays a part in the regulation and maturation of dendritic cells, and also improves their interaction with CD4+ T lymphocytes. It is involved in the functioning of NK cells and in promoting their activity through a modulation of NO levels. The decrease in NO production generated by this vitamin also results in COX-2 inhibition and PGE2 downregulation [87]. An additional anti-inflammatory mechanism performed by vitamin E is the inhibition of protein kinase C (PKC), which affects monocyte and neutrophil proliferation, together with a reduction in superoxide-free radical production by these cells. Besides, supplementation has demonstrated to improve phagocytosis and chemotaxis of neutrophils and macrophages in the elderly [31,38].

As for adaptive immunity, vitamin E improves mitogen-induced lymphocyte proliferation, enforces antibody response by B cells (including response to vaccination, especially in elderly subjects), enhances naive T cells immune synapse formation, starts T cell activation signals, and modulates Th1–Th2 balance. Supplementation restores IL-2 production, which improves the proliferation of T cells and general immune system functioning [87]. As a matter of fact, an improvement in Th1 cytokine response was observed in mice infected by influenza virus after vitamin E supplementation, which reduced their lung-related pathology and mortality [15].

All the mechanisms mentioned above support the hypothesis that vitamin E deficiency may increase susceptibility to pathogens. In animal models, this situation has also reported an increase in genetic mutations that promote virulence of several pathogens, including influenza virus and coronavirus. However, deficiency is rather uncommon in humans, even though it can occur in a secondary way owing to an intestinal malabsorptive disorder [15,31]. 

#### 3.5.2. Evidence about Vitamin E Regarding Respiratory Infections and COVID-19

Even though the role of vitamin E in the prevention of infectious diseases, such as influenza, has been considered and discussed, well-controlled human trials are still lacking [15]. A recent study in which adult volunteers received either tocopherol or tocotrienol supplementation showed a rise in the expression of several genes related to immunity, although the specific affected genes were different in each group [91].

Aging is related to a declination of immune response, leading to a greater predisposition to inflammation and oxidative stress. As a result, the risk of contracting respiratory infections in the elderly is bigger compared to general population, and their consequences are more severe. This is why vitamin E supplementation in this group of people seems to be particularly beneficial, so they can take advantage of it in order to counteract immunosenescence and improve their resistance to infection [92]. Vitamin E supplementation in the elderly was associated with an important reduction in re-hospitalization rate within 90 days after a first hospitalization due to pneumonia [93]. The existence of a negative association between plasma vitamin E levels has also been demonstrated, as well as a risk of infection in healthy adults over 60 years [38]. Furthermore, in another study conducted among elderly smokers, administration of this vitamin for 5–8 years reduced pneumonia incidence by 69% [92]. 

Nevertheless, results appear to be contradictory in some way, as some studies have reported a decrease in the risk of upper (but not lower) respiratory tract infections in the elderly due to vitamin E supplementation, though others have not observed a significant effect on the incidence, duration, or severity of infections in this group of population [94]. In consequence, the actual usefulness of vitamin E supplementation still remains unclear. Nowadays, there is a lack of clinical evidence pointing out a clear and actual benefit of vitamin E supplementation in SARS-CoV-2 infection. However, it has been suggested that a combination of vitamin E and C might be useful as an antioxidant therapy in COVID-19, especially in order to avoid cardiac complications. The RDA of α-tocopherol for healthy adults is 15 mg/d. In spite of the unlikelihood of deficiency, supplements of 50 mg/d have demonstrated to reduce pneumonia incidence, by always taking into consideration the possible pro-oxidant effect associated to excessive levels. So as to clarify the usefulness of this vitamin specifically in SARS-CoV-2 infection, either with a prophylactic or a therapeutic purpose, further investigation should be carried out, also with the aim of establishing a proper dosage for this intervention [15,46,95].

### 3.6. The Role of B Vitamins

#### 3.6.1. B Vitamins Mechanisms of Action

**Figure 6:**
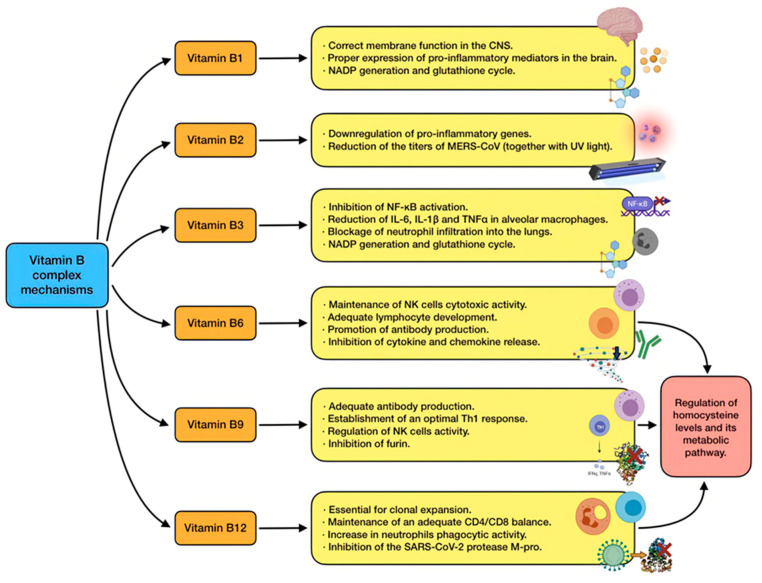
Summary of the main vitamin E mechanisms of action.

This group of water-soluble vitamins play an important role in cell metabolism. Despite their chemically distinct structures, they were grouped due to their similar ability to operate as coenzymes involved in many energy-related enzymatic processes [37]. The interest of B vitamins in SARS-CoV-2 patients is mainly based on indirect data [96]. The main mechanisms of action of B vitamins are shown in Figure 6.

B vitamins have reported to be efficient in strengthening antiviral responses and in lowering inflammation caused by viruses. Studies suggest that these vitamins may mediate interaction between immune cells and also regulate cytokine production, modifying the pathophysiological pathways of infections. In some cases, their immune activity is similar, but some of them present particular roles, associated with both innate and adaptive responses [15,31].

Vitamin B1: thiamine deficiency in the nervous system alters membrane function due to an impairment in fatty acid and cholesterol synthesis. With regard to immunity, its deficiency produces overexpression of some pro-inflammatory mediators in the brain, including COX-2, IL-1, IL-6, and TNFα. This may cause neuroinflammation, neuronal damage, and even neuronal cell death in the central nervous system, possibly leading to Wernicke–Korsakoff syndrome [31]. Both thiamin and niacin (vitamin B3) are needed for NADPH generation and glutathione cycle, which constitutes an important antioxidant pathway [97].

Vitamin B2: riboflavin has some immunomodulatory properties, and its deficiency has been associated with an upregulation of pro-inflammatory genes [31].

Vitamin B3: niacin is able to inhibit NF-κB activation, just as to reduce IL-6, IL-1β, and TNFα in alveolar macrophages. Likewise, it has also proved to block inflammation and neutrophil infiltration into the lungs in mice undergoing mechanical ventilation, and reduced ventilator-induced lung injury [98].

Vitamin B6: pyridoxine has direct implications in the immune system. It is essential in order to maintain cytotoxic activity of NK cells, antibody production by B cells, and adequate lymphocyte development. The vitamin also plays a part in the inhibition of cytokine and chemokine release [53]. Suboptimal consumption is related to altered lymphocyte maturation, lower quantities of circulating lymphocytes, and impaired antibody response, leading to attenuated humoral and cell-mediated immunity. Deficiency also causes spleen and thymus atrophy, and, as a result, affects T cell proliferation and immune response [38,53].

Vitamin B9: folate is commonly renowned for its role in one-carbon metabolism pathway, nucleic acid and amino acid metabolism, and DNA methylation. Nevertheless, this vitamin is also crucial for an adequate antibody production and an optimal Th1 response [53]. Suboptimal levels have been associated with imbalances in T-cell-mediated immune response, alterations in NK cell activity, and the appearance of panhypogammaglobinemia. Its deficiency also causes thymus and spleen atrophy, leading to reduced lymphocyte proliferation in them. In short, low levels of this vitamin produce a status of combined immunodeficiency [31].

Vitamin B12: multiple studies have reported the immunomodulatory effects of cobalamin. For example, it is essential for clonal expansion, so its deficiency (particularly frequent in the elderly due to lower absorption) is associated to lessened levels of plasma lymphocytes [53]. Suboptimal intake is also related to reduced levels of CD8+ T cells with an abnormally high CD4–CD8 ratio, together with attenuated NK cells activity. In addition, the phagocytic activity of neutrophils is also decreased by low serum levels of this nutrient [38].

In order to find effective molecular models against SARS-CoV-2, in silico studies have been carried out by taking into consideration the previously gathered information about SARS-CoV and MERS-CoV treatment. This virtual screening had the goal of identifying compounds capable of acting against the two proteases encoded by coronavirus polyprotein, which are needed for the processing and subsequent release of viral proteins. They are the 3-C like protease (M-pro) and a papain-like protease (Pl-pro). Using the crystal structure of these proteins, several existing drugs were screened so as to verify if they could be used to fight against the infection. According to the results, and based on lipophilic and hydrogen bonding interactions and ligand efficiency, vitamin B12 was the fourth molecule with the most powerful docking score against M-pro. These computational tools are particularly useful in COVID-19 crisis in order to find new therapeutic options, considering that effective treatments are still very limited [99]. However, these findings are based on a cell-free environment and does not measure efficiency. In addition, there are now suggestions pointing out that cobalamins are not really able to inhibit the protease [100].

An additional mechanism which involves more than one B vitamin is associated with an alteration in homocysteine metabolism. Homocysteine is considered a marker of oxidative stress, and it usually appears during vitamin B (especially folate and cobalamin) deficiency. Its metabolic pathway disruption has been proposed as one of the mechanisms involved in the death of cells infected by SARS-CoV-2 [15].

This is why a proper supply of vitamin B6, B9, and B12 in patients with COVID-19 is being taken into account as part of their treatment, in an attempt to control this negative feature. Administration of high doses of B-group multivitamins for prolonged periods has demonstrated to produce a significant improvement in homocysteine plasma levels [101].

#### 3.6.2. Evidence about B Vitamins Regarding Respiratory Infections and COVID-19

The available evidence on this topic concerning B vitamins is still very limited, yet there are some findings that deserve to be mentioned. Vitamin B1 has demonstrated its relevance when administered to critically ill patients, reducing mortality rates in those with septic shock [95]. Of note, there are a number of trials which show the benefits of a combination of thiamine, vitamin C, and hydrocortisone during sepsis, thus improving organ failure, severe pneumonia, time for shock reversal, and mortality rates [102]. As for vitamin B2, it has also demonstrated that, employed in vitro together with UV light, is able to reduce the titers of MERS coronavirus titers in human plasma below the limit of detection [103].

There is an inverse relationship between vitamin B6 intake and inflammatory signaling, which has been widely reviewed before. However, a clear correlation for this nutrient with epidemiologic COVID-19 indicators has not been found [104]. With regard to vitamin B6, in spite of the scarce scientific evidence available, it could possibly have a role in COVID-19 management. It has been proposed that folate may participate in the inhibition of furin, a convertase involved in spike protein cleavage, helping prevent the access of SARS-CoV-2 to host cells. Nevertheless, although suboptimal status positively correlates with COVID-19 incidence and mortality, this correlation is not as significant as the one found with vitamin D and C [53]. Finally, it has been reported that some of the countries less affected by the COVID-19 pandemic are the ones with the highest cobalamin intake. Likewise, those countries with an intake lower than the median are also the ones with a higher case and death rates. Supplementation with complex B vitamins, if considered, should be taken into account to basically revert a possible situation of deficiency, especially when it comes to B12 (whose RDA is 2.4 µg/d for adults and 4.5 µg/d during pregnancy) [53].

## 4. Conclusions

There is solid scientific evidence related to COVID-19, similar respiratory infections and ARDS development, indicating that vitamins might play an important role preventing and improving the progression of the disease. Vitamin D would have an essential role in SARS-CoV-2 infection, due to several mechanisms, including its interaction with the renin–angiotensin system. In this sense, the relation between inadequate vitamin D status, low sun exposure, and increased disease severity (especially in people at greater risk of deficiency such as the elderly and darker skin individuals) deserves to be highlighted. Vitamin C could also be beneficial by taking into account its antioxidant properties and its potential role in ameliorating URTIs, together with its use in ARDS and sepsis through a high-dose intravenous therapy. Less clear are the possible roles of vitamin A, E, and B. Nevertheless, they could act as adjuvants by a combination of several mechanisms associated with immune response and oxidative stress which support further investigation in relation to their utility in disease management. Therefore, vitamins might represent a relevant strategy for both prophylaxis and treatment of the infection during these –times. There is a lack of completed and well-designed clinical trials that conclusively demonstrate a role and benefits for supplementation during the infection by SARS-CoV-2. Finally, this adjuvant therapy could be easily applicable, not only to prevent nutritional deficiencies but also to boost the immune system and reduce inflammatory signaling and oxidative stress.
